# Diets of giants: the nutritional value of sauropod diet during the Mesozoic

**DOI:** 10.1111/pala.12385

**Published:** 2018-07-11

**Authors:** Fiona L. Gill, Jürgen Hummel, A. Reza Sharifi, Alexandra P. Lee, Barry H. Lomax

**Affiliations:** ^1^ School of Earth & Environment University of Leeds Leeds LS2 9JT UK; ^2^ Department of Animal Sciences University of Goettingen Goettingen Germany; ^3^ The School of Biosciences The University of Nottingham Sutton Bonington Campus, Sutton Bonington Leicestershire LE12 5RD UK

**Keywords:** Mesozoic, sauropod, diet, atmospheric CO_2_, metabolizable energy, carrying capacity

## Abstract

A major uncertainty in estimating energy budgets and population densities of extinct animals is the carrying capacity of their ecosystems, constrained by net primary productivity (NPP) and its digestible energy content. The hypothesis that increases in NPP due to elevated atmospheric CO_2_ contributed to the unparalleled size of the sauropods has recently been rejected, based on modern studies on herbivorous insects that imply a general, negative correlation of diet quality and increasing CO_2_. However, the nutritional value of plants grown under elevated CO_2_ levels might be very different for vertebrate megaherbivores than for insects. Here we show plant species‐specific responses in metabolizable energy and nitrogen content, equivalent to a two‐fold variation in daily food intake estimates for a typical sauropod, for dinosaur food plant analogues grown under CO_2_ concentrations spanning estimates for Mesozoic atmospheric concentrations. Our results potentially rebut the hypothesis that constraints on sauropod diet quality were driven by Mesozoic CO_2_ concentration.

Many Mesozoic terrestrial ecosystems were dominated by sauropod dinosaurs (Farlow *et al*. 2010; Sander *et al*. 2011; Barrett 2014) some of which reached over 50 metric tons in body mass during the Jurassic and Cretaceous (Sander *et al*. 2011). Global Mesozoic climate was very different to that of the present day, with equable temperatures, a low tropic to pole heat gradient with little polar ice (Frakes & Krassay 1992) and elevated atmospheric CO_2_ concentrations. A recent compilation of CO_2_ estimates (Foster *et al*. 2017) based on data from palaeosol, stomata, liverwort and alkenone proxies estimated Mesozoic carbon dioxide levels of between 229 (Late Cretaceous) and 2132 ppm (Late Triassic) compared to the present (~400 ppm) (Cerling 1991, 1992; Andrews *et al*. 1995; Ghosh *et al*. 1995; Beerling *et al*. 1998; McElwain 1998; Ekart *et al*. 1999; Lee 1999; Lee & Hisada 1999; McElwain *et al*. 1999; Chen *et al*. 2001; Ghosh *et al*. 2001, 2005; Tanner *et al*. 2001; Beerling & Royer 2002; Nordt *et al*. 2002, 2003; Robinson *et al*. 2002; Greenwood *et al*. 2003; Tabor *et al*. 2004; Haworth *et al*. 2005; McElwain *et al*. 2005; Prochnow *et al*. 2006; Sandler 2006; Sun *et al*. 2007; Cleveland *et al*. 2008; Fletcher *et al*. 2008; Beerling *et al*. 2009; Leier *et al*. 2009; Passalia 2009; Quan *et al*. 2009; Retallack 2009; Yan *et al*. 2009; Barclay *et al*. 2010; Bonis *et al*. 2010; Doria *et al*. 2011; Schaller *et al*. 2011; Steinthorsdottir *et al*. 2011, 2016; Wan *et al*. 2011; Gutierrez & Sheldon 2012; Hong & Lee 2012; Huang *et al*. 2012, 2013; Schaller *et al*. 2012, 2015; Mortazavi *et al*. 2013; Franks *et al*. 2014; Li *et al*. 2014; Ludvigson *et al*. 2015; Mays *et al*. 2015; Nordt *et al*. 2015; Steinthorsdottir & Vajda 2015; Whiteside *et al*. 2015; Du *et al*. 2016; Naafs *et al*. 2016; Wu *et al*. 2016). Both temperature and atmospheric CO_2_ can have a profound effect on vegetation and net primary productivity (Beerling & Woodward 2001), although other authors have questioned the assumption that CO_2_ is the most important driver of plant growth (e.g. Körner 2015). It has been postulated that sauropod gigantism was related to food availability and quality during the Mesozoic, but the nature of this relationship has been disputed.

Burness *et al*. (2001) speculated that increases in net primary production (NPP) as a function of elevated Mesozoic atmospheric CO_2_ levels was an enabling factor allowing sauropods to achieve their unprecedented size. This hypothesis was rejected by Midgley *et al*. (2002), who posited that light, water and nutrients would be limiting factors to increasing NPP even under high atmospheric CO_2_ concentrations. Outside of direct effects of CO_2_ on plant growth (i.e. NPP) there are thought to be indirect CO_2_ effects on the digestibility and nutritional value of plant material and it is this latter hypothesis we explicitly test in this study. This is because numerous authors (e.g. Midgley *et al*. 2002; Sander *et al*. 2011; Wilkinson & Ruxton 2013; Barrett 2014) have suggested that even if NPP did increase under elevated atmospheric CO_2_ levels, the poor nutritional quality of Mesozoic fodder, either as an inherent trait of non‐angiosperm plants (Weaver 1983) or due to low nitrogen content as a result of growth under elevated CO_2_ (Midgley *et al*. 2002; Wilkinson & Ruxton 2013), may have resulted in sauropods being obliged to consume vast quantities of food, necessitating large body size. Much evidence cited by those authors focuses on the response of angiosperms to CO_2_ growth concentrations up to twice current ambient levels and the effect on associated insect herbivores (e.g. Roth & Lindroth 1995; Curtis 1996; Wand *et al*. 1999; Ehleringer *et al*. 2002; Körner 2004). However, non‐angiosperm flora formed the bulk of sauropod diet; Mesozoic atmospheric concentrations of CO_2_ at times significantly exceeded twice current ambient levels, and the physiology, digestive processes and metabolism of vertebrate megaherbivores differ greatly from those of insects (Karasov & Douglas 2013). Consequently, the results of these insect feeding trials may not be directly transferable to understanding food quality for sauropods.

Alongside higher nitrogen:energy requirements, a critical difference between insects and sauropods is that the majority of insect herbivores use cell contents, including non‐structural carbohydrates such as starches and sugars, as their primary food source, whereas vertebrate megaherbivores utilize cell wall material (i.e. structural carbohydrates such as cellulose) as a food source, via fermentation by gut microbes (Karasov & Douglas 2013). Multiple lines of evidence indicate that sauropod dinosaurs also depended on gut microbes for fermentation of plant material in their diet (Farlow 1987; Hummel & Clauss 2011; Sander *et al*. 2011) therefore *in vitro* fermentation experiments represent an alternative approach to investigating food quality for plants grown under elevated CO_2_ (Akin *et al*. 1995; Carter *et al*. 1999; Morgan *et al*. 2004; Muntifering *et al*. 2006).

Combining *in vitro* fermentation experiments with plant growth experiments at CO_2_ concentrations representing Mesozoic atmospheric estimates provides a novel mechanistic framework to evaluate the nutritional value of sauropod food plants and test the competing theories of food quality and sauropod gigantism (Weaver 1983; Burness *et al*. 2001; Midgley *et al*. 2002; Sander *et al*. 2011; Wilkinson & Ruxton 2013; Barrett 2014). We emphasize that the experiments reported here were designed to address the knowledge gaps mentioned above, specifically in the plant species selected (dinosaur food plant analogues, predominantly gymnosperms and monilophytes rather than angiosperms), in the range of CO_2_ growth concentrations used, reflecting current consensus on Mesozoic atmospheric CO_2_ concentration estimates, and in the method of evaluating nutritional value, here represented by metabolizable energy and nitrogen content. Inevitably, our experiments have inherent limitations and represent a gross simplification of reality, but we believe that they can still contribute to the understanding of sauropod food quality during the Mesozoic.

## Material and method

### Plant growth experiments

Understory plants were chosen as models representative of the Mesozoic community (Gill *et al*. 2018, SI 1.1) with *Polypodium vulgare* (a fern) and *Equisetum hyemale* (a horsetail) representing a pre‐Cretaceous monilophyte flora (Pryer *et al*. 2004) and *Ranunculus acris* used as an analogue to some of the earliest Cretaceous herbaceous angiosperms, due to the antiquity of the family (Crane *et al*. 2000; Friis *et al*. 2010). Canopy plants are living examples of plants from groups with a well characterized fossil record, namely the gymnosperms *Metasequoia glyptostroboides*,* Gingko biloba* and *Araucaria araucana* (Lu *et al*. 2014). The CO_2_ growth concentrations were selected to span the range of recent estimated CO_2_ values for the Mesozoic (Foster *et al*. 2017).

All experiments were conducted in two walk‐in growth room chambers (UNIGRO, UK) meaning two [CO_2_] treatments could be run concurrently. Complete air exchange within each cabinet occurred three times per hour ensuring a fully mixed atmosphere. The first sets of experiments were conducted on the three understorey species, the angiosperm *R. acris* and the monilophytes *P. vulgare* and *E. hyemale*. The understorey species were grown for three months under treatment. Firstly, these species were grown at 400 and 1200 ppm CO_2_ for three months (May–August 2012). *R. acris* was grown from seed that germinated under treatment; *P. vulgare* and *E. hyemale* were placed under treatment as 3–4 month old plants. Secondly the growth chambers were reprogrammed to 800 and 2000 ppm CO_2_ and the process repeated with fresh plants (August–November 2012). All canopy species were placed under treatment as small 3–5 month old seedlings for a period of 6 months. The potting medium used for all plants was Levington M3. The canopy species were initially grown at 800 and 2000 ppm CO_2_ (July–December 2013) and then new plants were sourced and the process repeated with the growth rooms reprogrammed to 400 and 1200 ppm (January–July 2014). All other growth conditions remained constant. Plants received 10 hours of light (300 μmol/m^2^/s) per day in a simulated day/night program. Night temperature was set at a high of 17°C and daytime temperature peaked at 20°C for the understory and 22°C for the canopy species. Relative humidity was set at 70%. Throughout the experimental programme set points were monitored and there was minimal deviation from these set points. In all cases plants were kept well‐watered throughout their experiment and no additional fertilizer was applied due to the relatively short growth period of the experiment. Visual inspection of the plants throughout the experimental treatment indicated that plants were not under nutrient stress. Extensive ecophysiological measurements taken throughout the experiment and prior to harvest (Lee 2015) also showed no evidence of plant stress. In all cases plant material harvested and used in the analysis had developed in the target CO_2_ atmosphere.

Work in growth chambers is by necessity a trade‐off between facilities and time available to undertake the experiments. Ideally CO_2_ treatments would be repeated or run in parallel in a number of different chambers to fully test for chamber effects; effects driven entirely by the chamber which are independent of growth conditions (Porter *et al*. 2015). Whilst this approach is feasible when comparing two different CO_2_ concentrations this approach would quickly become untenable when comparing multiple species across multiple CO_2_ treatments. As an alternative, we have focused on within‐chamber plant replication and switching chambers for CO_2_ treatments. An additional limitation is the relatively short duration of the experiments, which we have acknowledged in the interpretation of our results.

At the end of the experimental run (three months for understory plants and six months for canopy species) leaves of the experimental plant material that had developed in the target CO_2_ concentration were harvested and then dried at 60°C. The duration of the understory versus the canopy experiments was different due to understory plants reaching maturity (either the production of flowers in the case of *R. acris* or spore production in *P. vulgare*;* E. hyemale* was also harvested at this time to allow for comparison) sooner than the canopy plants.

Leaves from individual plants (five plants per species per CO_2_ treatment) under investigation were harvested, pooled and after drying were milled to ~1 mm. From this bulk sample three aliquots were taken for the *in vitro* fermentation experiments described below.

### *In vitro* fermentation experiments

Hohenheim Gas Test equipment was used for *in vitro* evaluation of the digestibility of plant samples. Briefly, milled plant samples were incubated with cattle rumen fluid in gas‐tight syringes at 37°C (Menke & Steingass 1988). Gas production techniques are widely applied in agricultural feed evaluation and the strong relationship between digestibility and gas production is due to the direct stoichiometric linking of the production of gas and short chain fatty acids (SCFA) (Blümmel *et al*. 1999). The latter are the major end products of gut microbial fermentation and represent the major energy source for the host animal. The experiments were conducted over 72 h in order to reliably estimate the fermentation parameters (*a* + *b*;* k*). The length of the experiment also reflects the long ingesta retention time inferred for sauropod dinosaurs (Sander *et al*. 2011). Gas readings were taken at 4, 8, 12, 24, 32, 48, 56 and 72 h.

The fermentation parameters *a* + *b* (maximal gas production) and *c* (fractional fermentation rate) were estimated using the model:

$$\text{GP}=a+b\times (1-e^{-c\times t})$$

with GP being gas production at time *t*,* a* + *b* being maximal gas production (mL/200 mg DM) and *c* being the fractional fermentation rate (per h) (Blümmel & Oerskov 1993).

### Metabolizable energy estimation

Metabolizable energy (ME) was estimated from a linear regression set up from a data set of 24 h GP and ME contents of 24 feeds, based on data from (Menke & Huss 1987):

$$\begin{array}{cc} \text{ME}\;[\text{MJ}/\text{kg DM}] & =0.1474\times 24\;\text{h GP}[\text{mL}/200\;\text{mg DM}]\\ & \;+2.6412(R^{2}=0.8154) \end{array}$$

### Nutrient analyses

Neutral detergent fibre (NDF) and acid detergent lignin (ADL) analyses were done according to official German recommendations (values expressed without residual ash) (VDLUFA 2012). Both NDF and ADL were analysed using the Ankom fibre bag technique (Ankom Technology, Macedon, NY, USA).

Carbon and nitrogen analysis was carried out using a FLASH EA1121 CNS analyser (Thermo Scientific; https://www.thermofisher.com). Three ~20 mg subsamples of the pooled leaf material were analysed per species per CO_2_ treatment. Percentages of carbon and nitrogen were determined for each treatment and C:N ratio was calculated from these values.

### Statistical analyses

Effects of plant type and CO_2_ concentration on fermentation parameters (*a* + *b*;* c*) were evaluated via a two‐factorial anova (Gill *et al*. 2018, SI 2.1) followed by comparison of means by the Tukey–Kramer method, using SAS software (Gill *et al*. 2018, SI 3–5). The Tukey–Kramer method was also applied to evaluate differences in mean % N within taxa (Gill *et al*. 2018, SI 6).

To estimate ME of individual taxa grown under estimated Mesozoic atmospheric CO_2_ concentrations (Foster *et al*. 2017), polynomial regression equations were applied to the data (Gill *et al*. 2018, SI 7).

## Nitrogen

### Results

**Figure 1:**
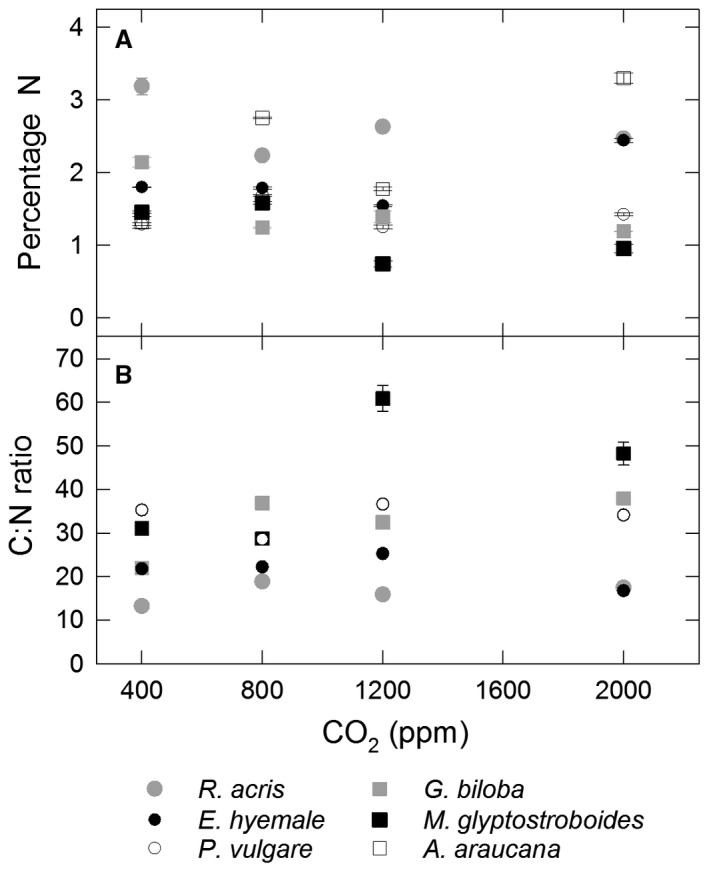
A, % N content; B, C:N content of experimental taxa at experimental CO_2_ growth concentrations. Note symbols for *E. hyemale* and *P. vulgare* are of a different size to enable all data points to be displayed. C:N ratio data for *A. araucana* not available. Where error bars are not shown, error is within the display of the symbol.

Changes in nitrogen content for the six species investigated did not show any clear trends with increasing CO_2_ growth concentration (Fig. 1A; Gill *et al*. 2018, SI 6). When comparing plants grown at 400 ppm CO_2_ and 800 ppm CO_2_ (comparable to the modern ambient and twice‐ambient values used in the majority of published studies) two of the six experimental taxa, *R. acris* and *G. biloba*, showed a significant reduction in % N content; *A. araucana* and *P. vulgare* showed a significant increase in % N; and the % N content of *E. hyemale* and *M. glyptostroboides* was not significantly different between the two treatments (Fig. 1A; Gill *et al*. 2018, SI 6). When comparing N content between ‘ambient’ (i.e. 400 ppm) and CO_2_ growth concentrations higher than twice‐ambient (i.e. our 1200 and 2000 ppm growth treatments), differences were observed for some taxa. *Metasequoia glyptostroboides* showed a notable decrease in % N at higher CO_2_ growth concentrations (Gill *et al*. 2018, SI 6) and a two‐fold increase in C:N ratio for the 1200 ppm treatment compared to the 400 ppm and 800 ppm CO_2_ treatments (Fig. 1B). The % N value for *E. hyemale* was significantly higher for plants grown under 2000 ppm CO_2_ than for all other CO_2_ concentrations.

### Discussion

Few published data exist on carbon and nitrogen content under ambient or elevated CO_2_ growth conditions for our experimental species. Hummel *et al*. (2008) measured crude protein content of a number of plant species from ambient conditions, including *G. biloba* and *Equisetum* spp., from which % N can be derived (crude protein is calculated as % N × 6.25). Our results for plants grown under the ambient‐equivalent treatment (i.e. 400 ppm CO_2_) are similar to those of Hummel *et al*. (2008), with our *G. biloba* leaves having 2.14% N vs 2.5% N for those of Hummel *et al*. (2008) and our *E. hyemale* having 1.80% N vs 1.9% N for *Equisetum* spp. from Hummel *et al*. (2008). Similarly, Decherd (2006) measured 2.4% N and a C:N ratio of 21.4 in *G. biloba* leaves grown under ambient CO_2_ concentration (370 ppm) and 1.3% N and a C:N ratio of 40.3 in *G. biloba* leaves grown under 2000 ppm CO_2_ (both treatments with ambient O_2_). For comparison, our *G. biloba* % N was 1.19 for plants grown under 2000 ppm CO_2_ and our C:N ratios were 22 for plants grown under 400 ppm CO_2_ and 38 for plants grown under 2000 ppm CO_2_. Bacon *et al*. (2016) found a considerably lower mean % N for *G. biloba* of 0.68 for plants grown under ambient conditions (380 ppm CO_2_ and 20.9% O_2_) and 0.21 for plants grown under 1500 ppm CO_2_, with corresponding C:N ratios of 73 and 242.

The assumption that leaf % N content of Mesozoic flora would have been reduced and C:N ratios increased by elevated atmospheric CO_2_ concentrations (Midgley *et al*. 2002; Sander *et al*. 2011; Wilkinson & Ruxton 2013) is based on studies that focused largely on angiosperms (Roth & Lindroth 1995; Curtis 1996; Wand *et al*. 1999; Ehleringer *et al*. 2002; Körner 2004) with nitrogen data from only two non‐angiosperm species (*Pinus ponderosa* and *Pinus taeda*; Curtis 1996). These and other studies (e.g. Stiling & Cornelissen 2007) undoubtedly show an overall trend towards reduced leaf % N with elevated CO_2_ growth concentration, including the majority of the limited number of gymnosperm taxa studied, but some species deviate from this trend. For example, leaf nitrogen content was reduced in the angiosperm *Betula papyrifera* but not the gymnosperm *Pinus strobus* for plants grown at elevated (650 ppm) versus ambient (350 ppm) CO_2_ concentrations (Roth & Lindroth 1994). Similarly, a recent study investigating the effects of simulated palaeoatmospheres on non‐angiosperm plant growth (Bacon *et al*. 2016) found that one of the six species studied, *Nageia nagi*, had a lower C:N ratio and higher % N for plants grown under 1500 ppm CO_2,_ compared to those grown under ambient CO_2_ concentrations. These findings are consistent with our results and together these examples indicate that a reduction in leaf % N is not a universal consequence of growth under elevated CO_2_, at least for gymnosperms, but may be taxon‐specific. Our results also reinforce previous findings (e.g. Kaplan *et al*. 2012) that plant responses to moderately elevated CO_2_ may be different under superelevated CO_2_ concentrations. However, an important caveat when interpreting nitrogen data from both our study and those cited, is that all were based on growth experiments of relatively short duration: 35 days for Decherd (2006); 53 days for Roth & Lindroth (1994); 3 months for our understory plants; 6 months for our canopy plants; and 18 months for Bacon *et al*. (2016) (K. Bacon, pers. comm. November 2017). The short durations of these experiments mean that nitrogen in the growth medium is unlikely to be significantly depleted and become a limiting factor for growth, which may be the case in natural environments (e.g. Körner 2015).

As discussed above, changes in leaf nitrogen content or C:N values are not inevitable for plants grown under elevated CO_2_ concentrations, but if they did occur, sauropods may have responded differently to modern insect herbivores. The most fundamental difference between the insects in the studies cited (Roth & Lindroth 1994, 1995; Ehleringer *et al*. 2002) and sauropods, is the reliance of the former on cell contents (Abe & Higashi 1991) and the presumed reliance of the latter on microbial fermentation of cell walls (Farlow 1987; Hummel & Clauss 2011; Sander *et al*. 2011) to meet their nutritional needs. Phytophagous (*sensu* Abe & Higashi 1991) insects have low absolute energy requirements and high nitrogen requirements, which are met by cell contents (Karasov & Douglas 2013). They may therefore be particularly sensitive to decreases in leaf % N or increases in leaf C:N ratio. Megaherbivores have high absolute energy demands, which can be met by fermentation of abundant cell wall material, and may have metabolic (e.g. lower metabolic rates), physiological (e.g. larger body size) or behavioural (e.g. lower activity levels) adaptations to accommodate food resources with low leaf % N or high C:N ratio (e.g. Grubb 1992; Midgley 2005; Hummel *et al*. 2008). The few published studies applying the feeding trial approach to vertebrates (Wroblewitz *et al*. 2008; Habeck & Lindroth 2013), albeit with a limited number of taxa and CO_2_ concentrations, have shown no negative impact of food plant growth under elevated CO_2_.

The evidence presented here indicates that nitrogen content of food plants may not necessarily have been a limiting factor in sauropods’ use of plant resources during the Mesozoic. This does not exclude the possibility that it may have been a limiting factor for some taxa, but strongly suggests that the effect may be species‐specific.

## Metabolizable energy

### Results

**Figure 2:**
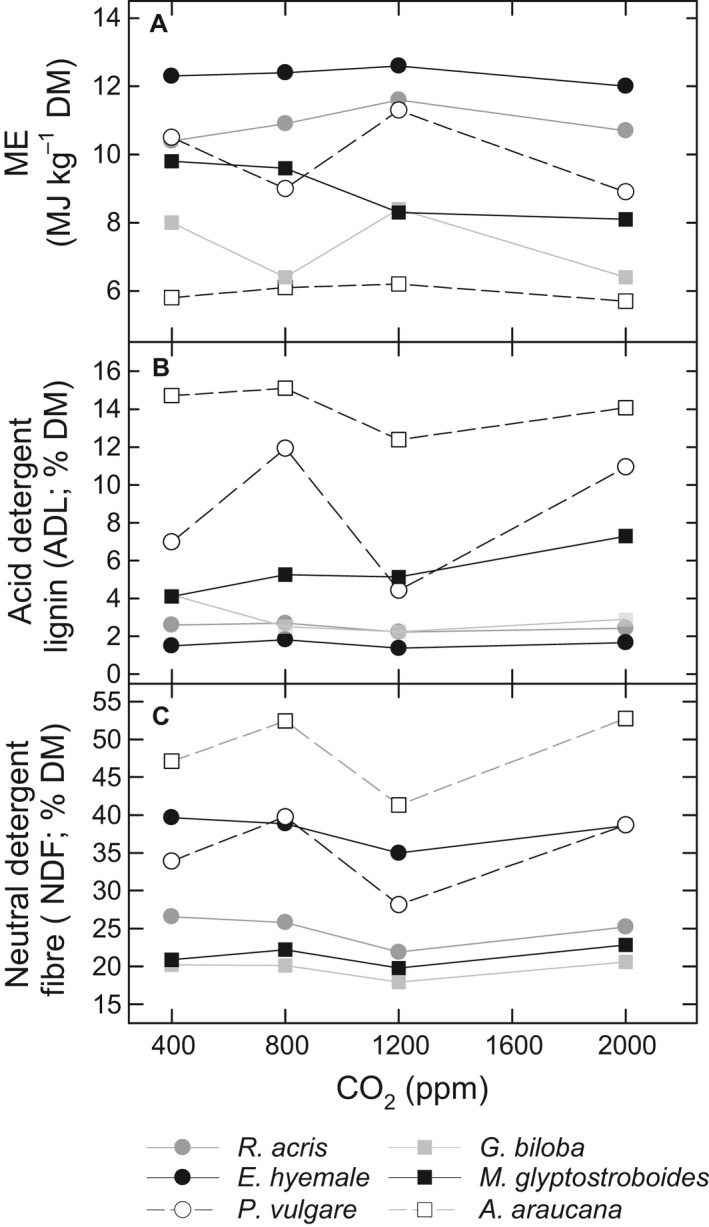
A, metabolizable energy (ME) content; B, acid detergent lignin (ADL) content; C, neutral detergent fibre (NDF) content of experimental taxa at experimental CO_2_ growth concentrations.

Figure 2 summarizes ME (estimated from gas production (GP) as described in Material and Method, above), neutral detergent fibre (NDF) and acid detergent lignin (ADL) values for each experimental taxon, under each CO_2_ growth concentration.

Plant species and CO_2_ growth concentration both significantly affected the rate and extent of GP (Gill *et al*. 2018, SI 2.1, 2.2) and hence ME content (Fig. 2A). *E. hyemale* had the maximum GP of the taxa investigated and *A. araucana* had the lowest overall cumulative GP (Gill *et al*. 2018, SI 2.1, 3.1). When comparing the mean GP across all taxa, the highest overall GP was associated with growth under 1200 ppm CO_2_ (Gill *et al*. 2018, SI 4). GP also varied significantly between plants of the same taxon grown at different CO_2_ concentrations for all experimental taxa except *E. hyemale* (although not necessarily between every CO_2_ growth concentration for each species) (Gill *et al*. 2018, SI 5). These results demonstrate the impact of taxon and CO_2_ growth concentration on the digestibility of plant matter by digestive tract microbes.

### Discussion

Our results show that the GP, and therefore ME content, of the two monilophyte species and one of the gymnosperm species tested (*M. glyptostroboides*) is comparable to (or exceeds in the case of *E. hyemale*) that of the angiosperm species evaluated, when comparing mean values across all CO_2_ treatments (Gill *et al*. 2018, SI 3). This is consistent with previous findings, albeit on plants grown under modern ambient CO_2_, demonstrating that the ME content of some potential dinosaur food plant analogues is not intrinsically low, but is comparable to angiosperm ME values in some cases (Hummel *et al*. 2008). Looking at each taxon individually, maximum ME (Fig. 2A) was estimated in plants grown at a CO_2_ concentration of 1200 ppm, for five of the six taxa evaluated, although this difference was statistically significant (based on measured gas production) only for *G. biloba* (Gill *et al*. 2018, SI 5). A corresponding decrease in NDF (i.e. hemicellulose, cellulose and lignin, also referred to as structural carbohydrates) and ADL at this CO_2_ concentration was observed (Fig. 2B, C), which may be due to increased production of easily‐fermented non‐structural carbohydrates, such as sugars, at the expense of structural carbohydrates. *Metasequoia glyptostroboides* exhibited a different response, with ME decreasing and ADL increasing with increasing CO_2_ growth concentrations. The negative correlation between ME and ADL has been observed in previous studies (Hummel *et al*. 2006), and is attributed to the fact that lignin is not only not fermentable by gut microbes in anaerobic environments, but also forms linkages to normally digestible structural carbohydrates, especially hemicelluloses, rendering them completely indigestible (Van Soest 1994). The same reasoning may also explain why *E. hyemale* has the highest overall ME, since it has very low ADL content, implying that the majority of structural carbohydrates are available for fermentation. The low lignin content of *E. hymale* may be due to use of silica as an alternative structural element, which has also been reported for Mesozoic examples of the genus (e.g. Channing *et al*. 2011).

**Figure 3:**
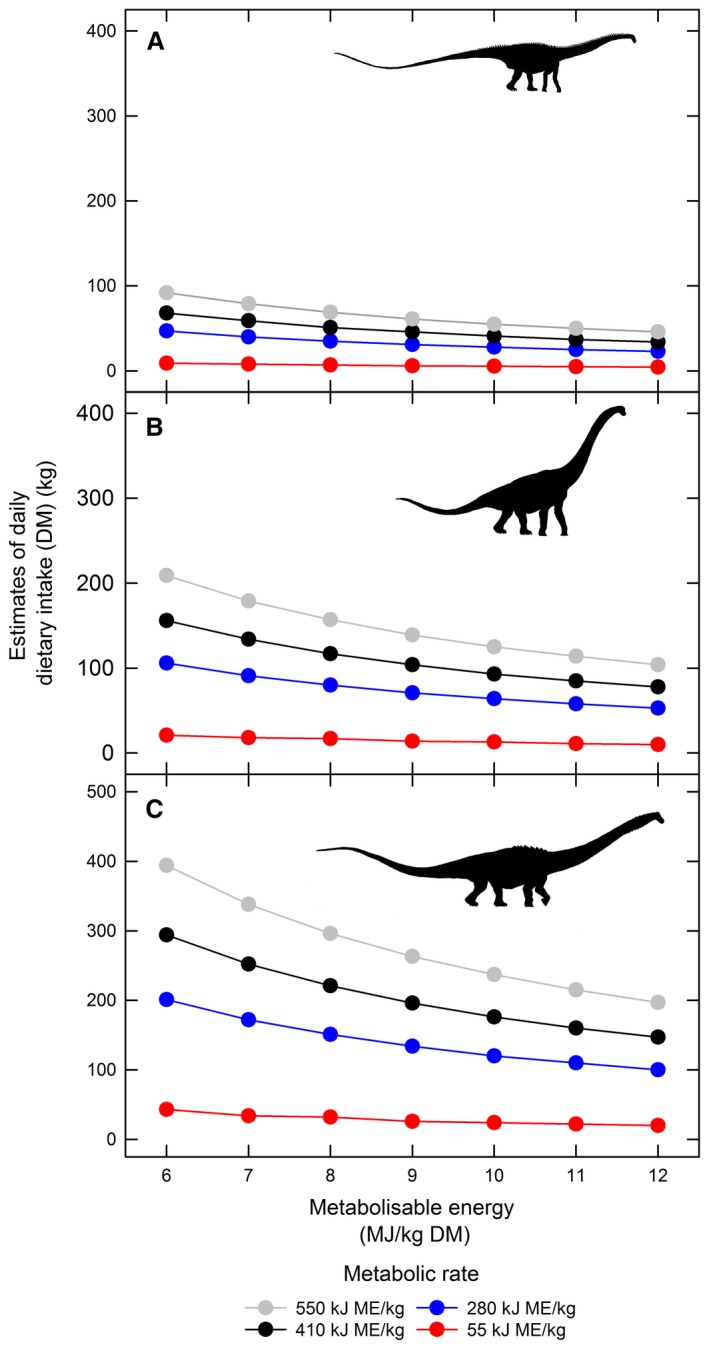
Daily food intake estimates at variable metabolizable energy content of food for: A, 10 t sauropod; B, 30 t sauropod; C, 70 t sauropod; assuming a metabolic rate of: 55 kJ ME/kg body weight^0.75^ (red, equivalent to modern day reptiles); 280 kJ ME/kg body weight^0.75^ (blue); 410 kJ ME/kg body weight^0.75^ (black); 550 kJ ME/kg body weight^0.75^ (grey, equivalent to modern day mammals) (Hummel *et al*. 2008). Body sizes were chosen to represent small, average and maximal sauropod body size illustrated in: A, by *Diplodocus* sp.; B, by *Brachiosaurus* sp.; C, by *Dreadnoughtus* sp. See Gill *et al*. (2018, S. I. 8.1) for details of calculations.

**Figure 4:**
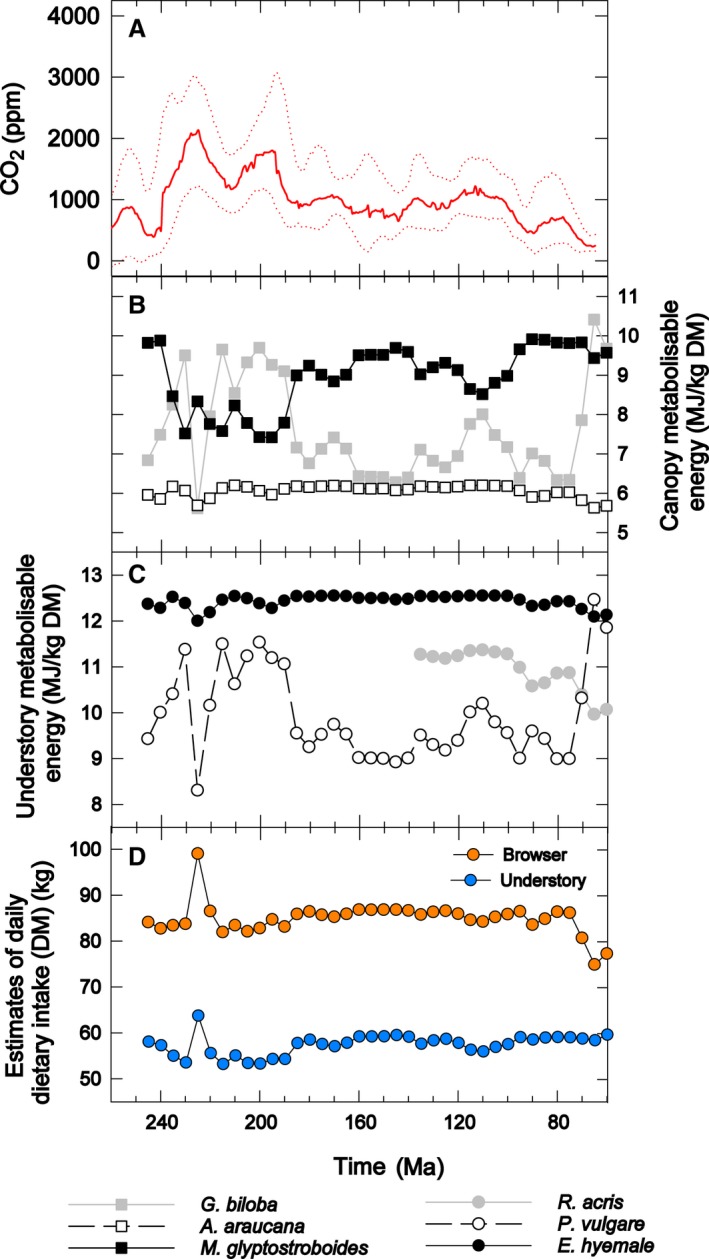
A, atmospheric CO_2_ estimates through the Mesozoic (Foster *et al*. 2017). B, estimated ME values for canopy experimental taxa throughout the Mesozoic. C, estimated ME values for understory experimental taxa throughout the Mesozoic. D, daily food intake estimates for hypothetical 30 t sauropod with a metabolic rate of 280 kJ ME/kg body weight^0.75^ during the Mesozoic with browsing (*A. araucana *+ *M. glyptostroboides *+ *G. biloba*) vs understory (*E. hyemale *+ *R. acris *+ *P. vulgare*) diet. See Gill *et al*. (2018, SI 8.3) for details of calculation.

Our experiments have demonstrated species‐specific responses, in terms of ME content, to growth under elevated CO_2_ concentrations (Fig. 2), which may have had significant implications for the amount of plant biomass needed to sustain terrestrial herbivores during the Mesozoic. We have modelled the effect of differing food ME content on estimated sauropod daily intake requirements for a range of sauropod body sizes and metabolic rates corresponding a typical modern reptile (55 kJ ME/kg BW^0.75^/day), a typical modern mammal (550 kJ ME/kg BW^0.75^/day) and two intermediate metabolic rates (Hummel *et al*. 2008; Fig. 3; Gill *et al*. 2018, SI 8.1). Using our experimental results, we estimate that a hypothetical 30 t sauropod with an energy requirement of 280 kJ ME/kg BW^0.75^/day (i.e. with a metabolism intermediate between modern lizards and mammals; Hummel *et al*. 2008) would need to eat 110 kg per day (all food intake estimates are reported on a dry matter (DM) basis for ease of comparison) of *A. araucana* grown under atmospheric CO_2_ concentrations of 2000 ppm, whereas an identical animal would need to eat less than half that amount, i.e. 51 kg/day, if feeding exclusively on *E. hyemale* grown under CO_2_ levels of 1200 ppm (Gill *et al*. 2018, SI 8.2). Extending this approach, we have modelled (Fig. 4) expected changes in ME as CO_2_ concentrations fluctuated through the Mesozoic (Fig. 4A; Foster *et al*. 2017). Figure 4B–C shows estimates of ME for the experimental taxa and Figure 4D indicates how broad resolution modelled variation in atmospheric CO_2_ may have impacted food intake for browsing/canopy‐feeding sauropods versus understory‐consuming sauropods throughout the Mesozoic (Gill *et al*. 2018, SI 8.3). Food intake estimates are higher for browsing/canopy feeding sauropods than for understory‐consuming sauropods throughout the Mesozoic, but the intake estimates for these two broad diet categories during the Mesozoic run largely in parallel, until the Late Cretaceous, when browser/canopy‐feeding intake estimates are substantially reduced, although still considerably higher than for understory eaters.

## Revised sauropod dinosaur biomass and population density in the Morrison formation

The Late Jurassic Morrison Formation is one of the most well‐known and widely studied dinosaur deposits, allowing us to translate and apply our theoretical approach to the fossil record. Niche partitioning between sauropod species in the formation has been suggested based on multiple lines of evidence (Farlow *et al*. 2010), including (but not restricted to) skull functional morphology (Button *et al*. 2014), carbon stable isotopic analysis of bones and teeth (Tütken 2011) and tooth replacement rates (D'Emic *et al*. 2013). For example, Morrison Formation *Diplodocus* sp. have been interpreted as low browsers, with a diet based on consuming monilophytes such as ferns and *Equisetum* sp., whereas Morrison Formation *Camarasaurus* sp. show evidence for higher browsing and a more mixed diet, with a higher proportion of woody, probably coniferous material (Button *et al*. 2014; Tütken 2011). Combining this palaeontological evidence with our experimental data allows us to estimate food intake for these two Morrison Formation sauropods, although we acknowledge the simplification inherent in using our limited range of relevant experimental species, in contrast to the known diversity of the Morrison Formation flora (Farlow *et al*. 2010). Again assuming an energy requirement of 280 kJ ME/kg BW^0.75^/day, a 10.8 t (Farlow *et al*. 2010) Morrison Formation *Diplodocus* sp. feeding exclusively on ferns would need to eat 33.2 kg per day (based on ME values derived from *P. vulgare*). The same animal feeding exclusively on *Equisetum* sp. would need to consume 23.8 kg/day (based on values derived from *E. hyemale*) or 27.7 kg/day of a 50:50 mixture of the two plant groups. Under the same assumptions, a 12.6 t (Farlow *et al*. 2010) *Camarasaurus* sp. with a mixed diet consisting of equal proportions of monilophyte understory and canopy plants (using values derived from *P. vulgare*,* E. hyemale*,* A. araucana*,* G. biloba* and *M. glyptostroboides*) would need to eat 34.2 kg/day. For comparison, a typical modern elephant, weighing 7 t, with an energy requirement of 550 kJ ME/kg BW^0.75^/day would need to eat 47.0 kg/day of *P. vulgare*, 33.7 kg/day *E. hyemale* sp., 39.3 kg/day of a 50:50 mixture of these two species or 48.4 kg/day of a diet consisting of equal parts of *P. vulgare*,* E. hyemale*,* A. araucana*,* G. biloba* and *M. glyptostroboides*. See Gill *et al*. (2018), SI 8.4 for details of calculations in this section.

Models of dinosaur biomass and population density usually do not consider variation in metabolizable energy content between different dinosaur food plants, and between the same taxa growing under different CO_2_ concentrations (e.g. Farlow 1976; McNab 2009; Trammer 2011) or, if they do, consider it at an extremely broad level, e.g. gymnosperms versus angiosperms (Midgley *et al*. 2002). At best, ME values from dinosaur food plant analogues grown at ambient CO_2_ concentration are used (Hummel *et al*. 2008; Farlow *et al*. 2010). However, we have demonstrated that ME varies with CO_2_ growth concentration for the majority of our experimental species and that the response to growth in varying levels of CO_2_ differs between species. Therefore, incorporating these factors into calculations may improve estimates. Returning to the Morrison formation and substituting our experimentally derived maximum (based on *E. hyemale*) and minimum (based on *A. araucana*) ME values for the Late Jurassic results in an estimated increase in carrying capacity from a maximum of 54 800 000 kJ/km^2^/day according to Farlow *et al*. (2010) to 68 500 000 kJ/km^2^/day (Gill *et al*. 2018, SI 8.5). Assuming that Morrison Formation herbivores found 50% of the plant matter available and palatable (Farlow *et al*. 2010), this results in a change in estimated population density of approximately 20%: from 6.4–1566 (Farlow *et al*. 2010) to 6.5–1954 individuals per km^2^ of landscape (Gill *et al*. 2018, SI 8.5).

## Conclusions

This study has employed a novel experimental approach towards estimating the nutritional value of dinosaur food plants during the Mesozoic, challenging existing assumptions about the relationship between CO_2_ levels, food quality and sauropod gigantism. In particular, our data clearly challenge a view of a constant and linear decrease of diet quality with increasing atmospheric CO_2_, which has previously been suggested as a driver for sauropod gigantism (Midgley *et al*. 2002; Wilkinson & Ruxton 2013). The mechanistic approach employed in this study could equally well be applied to other ecosystems and megaherbivore groups, for example, Miocene mammals (Janis *et al*. 2000).
